# Chitosan Derivative-Based Microspheres Loaded with Fibroblast Growth Factor for the Treatment of Diabetes

**DOI:** 10.3390/polym15143099

**Published:** 2023-07-20

**Authors:** Jue Wu, Qian Chen, Wenfei Wang, Yuhong Lin, Hong Kang, Zheng Jin, Kai Zhao

**Affiliations:** 1 College of Chemistry and Material Sciences, School of Life Science, Heilongjiang University, Harbin 150080, China; wujuekk@163.com (J.W.); chenqian48@sinopharm.com (Q.C.); kanghonghd@163.com (H.K.); 2 Bio-Pharmaceutical Lab, College of Life Sciences, Northeast Agricultural University, Harbin 150030, China; wangwenfei@neau.edu.cn; 3 Zhejiang Provincial Key Laboratory of Plant Evolutionary Ecology and Conservation, Taizhou Key Laboratory of Biomedicine and Advanced Dosage Forms, School of Life Sciences, Taizhou University, Taizhou 318000, China; yuhonglin930412@163.com

**Keywords:** sustained and slow release, drug delivery, chitosan derivative-based microsphere, diabetes mellitus, fibroblast growth factor

## Abstract

Diabetes mellitus type 2 (T2DM) is a disease caused by genetic and environmental factors, and the main clinical manifestation is hyperglycemia. Currently, insulin injections are still the first-line treatment for diabetes. However, repeated injections may cause insulin resistance, hypoglycemia, and other serious side effects. Thus, it is imperative to develop new diabetes treatments. Protein-based diabetes drugs, such as fibroblast growth factor-21 (FGF-21), have a longer-lasting glycemic modulating effect with high biosafety. However, the instability of these protein drugs limits their applications. In this study, we extract protein hypoglycemic drugs with oral and injectable functions. The FGF-21 analog (NA-FGF) was loaded into the chitosan derivative-based nanomaterials, N-2-Hydroxypropyl trimethyl ammonium chloride chitosan/carboxymethyl chitosan (N-2-HACC/CMCS), to prepare NA-FGF-loaded N-2-HACC/CMCS microspheres (NA-FGF-N-2-HACC/CMCS MPs). It was well demonstrated that NA-FGF-N-2-HACC/CMCS MPs have great biocompatibility, biostability, and durable drug-release ability. In addition to injectable drug delivery, our prepared microspheres were highly advantageous for oral administration. The in vitro and in vivo experimental results suggested that NA-FGF-N-2-HACC/CMCS MPs could be used as a promising candidate and universal nano-delivery system for both oral and injectable hypoglycemic regulation.

## 1. Introduction

Diabetes mellitus type 2 (T2DM) is a systemic and metabolic disease with hyperglycemia caused by genetic and environmental factors [1,2]. The long-term higher hyperglycemia of diabetic patients is mainly caused by the defect of insulin secretion or the impaired biological function of insulin (insulin resistance), which leads to the occurrence of serious complications and the dysfunction of the main organs [3,4,5]. Drug delivery systems mainly include injection administration and oral administration. Although good treatment outcomes have been obtained after injecting insulin as the primary treatment for diabetes, patient adherence is still largely limited due to the fear of factors such as injection [6,7,8,9]. In comparison with insulin injections, most of the oral hypoglycemic drugs on the market are chemical drugs. However, the long-term oral administration of these chemical drugs will accelerate the senescence and dysfunction of islet B cells in patients with type 2 diabetes [10,11,12]. Fibroblast growth factor-21 (FGF-21) is a member of the FGF family mainly expressed in the liver [13]. It has been found that the regulation mechanism of FGF-21 appears to be insulin-independent, and FGF-21 can be used as an additive to enhance insulin activity. In contrast to insulin, FGF-21 needs to be present in the cells for several hours to produce a robust response to glucose uptake, and this effect can be reduced by the protein synthesis inhibitor cycloheximide [14]. In addition, FGF-21 induces a significant upregulation of the insulin-independent glucose transporter GLUT1 in 3T3-L1 adipocytes [15]. This means that it can slowly lower blood sugar levels and avoid limitations such as insulin resistance compared to insulin injections. However, such proteins are at risk of poor stability, low drug-loading capacity, and a short half-life, which directly affect their clinical therapeutic effects. Therefore, developing an ideal oral protein delivery system with excellent biostability, bioavailability, and high loading ability is urgently needed [16,17,18].

In recent years, various kinds of nano-delivery systems have been developed for the effective delivery of protein drugs. Among them, liposomes are artificial membranes that wrap a drug in a structure similar to a lipid bilayer and form microvesicles. Owing to their high biocompatibility, low immunogenicity, and low drug-loading efficiency, liposomes have been widely used in drug delivery [19]. However, the size of the lamination and the surface properties of liposomes have a significant influence on their in vivo applications. Another common method of drug delivery is non-metallic nanomaterials, mainly silica nanomaterials. This material has the advantages of strong drug-loading capacity and surface modification. However, it easily accumulates in the body and exhibits toxicity in tissues [20].

Benefiting from the properties of great biocompatibility, biodegradability, bacteriostasis, and enhanced immunity, chitosan has exhibited great application prospects in the fields of food processing, bacterial inhibition, drug delivery, and tissue regeneration. Lisuzzo et al. used the electrostatic interaction between chitosan and halloysite nanotubes to prepare a drug-delivery system with khellin [21]. The incorporation of different amounts of nanohydroxyapatite into the chitosan matrix has created composite scaffolds with enhanced elasticity and flexibility [22]. Chitosan can also be used as an effective material for heavy metal regeneration adsorbents [23,24]. In addition, the construction of chitosan-based microspheres and nanoparticles through emulsive cross-linking or ionic cross-linking can be applied to the efficacious delivery of drugs with great sustained release effects [25,26,27,28]. On this basis, chitosan is considered a functional biomaterial with great application potential. However, the poor water solubility of chitosan largely limits its actual applications in biomedical research [29,30,31]. The hydroxyl and amino groups in the structure of chitosan can be easily modified to introduce different active groups, which endow chitosan with excellent biological functions [32]. For example, carboxymethylated chitosan and quaternary ammonium-functionalized chitosan significantly improve its water solubility, and acylated chitosan enhances the antibacterial property of chitosan [33,34,35,36]. Among them, N-2-Hydroxypropyl trimethyl ammonium chloride chitosan (N-2-HACC) and carboxymethyl chitosan (CMCS) are widely used in biology and biomedicine because of their good water solubility, biological adhesion, unique physicochemical properties, and biological functions.

In this study, biodegradable N-2-HACC/CMCS microspheres loaded with the FGF-21 analog (NA-FGF) (NA-FGF-N-2-HACC/CMCS MPs) were synthesized. The prepared microspheres could effectively prolong the half-life of NA-FGF to make it more durable. To investigate the bioactivity of NA-FGF-N-2-HACC/CMCS MPs, injection and oral administration were both employed, and the blood glucose level changes caused by NA-FGF-N-2-HACC/CMCS MPs in mice were observed. The constructed NA-FGF-N-2-HACC/CMCS MPs can be used as a novel drug agent to replace traditional insulin-based therapy in the treatment of type 2 diabetes and provide a theoretical basis for addressing insulin resistance.

## 2. Materials and Methods

### 2.1. Materials

Sodium dodecyl sulfate (SDS), N,N,N′,N′-Tetramethylethylenediamine (TEMED), chitosan, imidazole, streptozotocin (STZ), and isopropyl-β-D-thiogalactoside (IPTG) were obtained from Sigma-Aldrich Co., Ltd. (Saint Louis, MO, USA). Dulbecco’s modified Eagle’s medium (DMEM) was obtained from Invitrogen Co., Ltd. (Carlsbad, CA, USA). Powdered agar, tryptone, and glucose were obtained from Beijing Aaoboxing Biotechnology Co., Ltd. (Beijing, China). Cell Counting Kit-8 was purchased from Beijing Quanshijin Biotechnology Co., Ltd. (Beijing, China). NA-FGF gene-engineered strain DNZY1 was provided by the Pharmaceutical Engineering Laboratory, College of Life Sciences, Northeast Agricultural University (Harbin, China).

### 2.2. Biosynthesis and Characterization of the NA-FGF

NA-FGF protein was expressed in the *E.coli* expression system and purified with a Ni-NTA column. Briefly, Stain DNZY1 was cultured and bio-fermented in the LB medium. After being co-incubated with IPTG (0.25‰, *v*/*v*) for 4 h, the thallus was collected and washed by centrifugation at 4 °C, 4000 r/min, for 30 min. The supernatant was then purified through a Ni-NTA column, and the filtrate was dialyzed to obtain the NA-FGF protein. SDS-PAGE analysis was used to detect the purity of the obtained protein. The protein whose purity is greater than 95% can be used in subsequent experiments.

### 2.3. Establishment of the Diabetic Mouse Model

The C57BL/6 mice (3–4 weeks) were purchased from Shanghai Slake Experimental Animal Co., Ltd. (Shanghai, China) (SCXK 2022-0004). All the animal studies were approved by the Laboratory Animal Welfare and Ethics Committee, Taizhou University, China. The care of laboratory animals and all animal experiments were in accordance with the “National Research Council’s Guide for the Care and Use of Laboratory Animals”. To establish the diabetes model, 85 mice were injected with 100 mg/kg streptozotocin and fed 10% (*m*/*v*) glucose, respectively. Mice were fasted overnight before injection, resumed feeding after injection, given 10% glucose water, and then replaced with normal water after 72 h. After injection of STZ solution, the blood glucose values of fasting mice were measured at 2 d intervals. Modeling was considered successful once blood glucose levels were higher than 16.65 mmol/L.

### 2.4. Activity Analysis of the NA-FGF3

The diabetic mice were randomly divided into two groups of five mice each. The first group of mice was injected subcutaneously with saline, and the second group of mice was injected subcutaneously with NA-FGF. The injection dose was 1 mg/kg. Blood samples before and 1, 2, 3, 4, and 5 h after injection were collected from the tail vein, respectively, and blood glucose values were measured by a glucometer (YUWELL, Jiangsu, China).

### 2.5. Preparation and Characterization of NA-FGF-N-2-HACC/CMCS MPs

N-2-HACC and CMCS were synthesized as previously described and reported by Jin et al. [37]. On the basis of electrostatic interactions, the novel NA-FGF-N-2-HACC/CMCS MPs with uniform and stable particle sizes were prepared by using positive and negative ion self-assembly methods. The concentrations of N-2-HACC, CMCS, and NA-FGF for preparing NA-FGF-N-2-HACC/CMCS MPs were optimized by univariate tests of encapsulation efficiency (EE) and loading capacity (LC), respectively. The following Formulas (1) and (2) were used to calculate the EE and LC. m_0_ is the total mass of NA-FGF added, m_1_ is the mass of NA-FGF in the supernatant, and m is the total mass of drug-loaded NA-FGF-N-2-HACC/CMCS MPs. EE and LC were determined by the following equations:

(1) $$\mathrm{EE}\;(\%)=\frac{m_{0}-m_{1}}{m_{0}}\text{×}100\%$$

(2) $$\mathrm{LC}(\%)=\frac{m_{0}-m_{1}}{m}\text{×}100\%$$

Based on the optimal preparation conditions, 10 mL of 1.5 mg/mL N-2-HACC, 5 mL of 1.2 mg/mL CMCS, and 12.5 mL of NA-FGF were mixed and stirred at 1000 r/min for 1 h under protection on ice. The precipitate was then collected by centrifugation and washed three times; the supernatant was discarded, and the residues were lyophilized. The structure of prepared NA-FGF-N-2-HACC/CMCS MPs was characterized by Fourier transform infrared spectroscopy (FTIR) (NICOLET5700, Thermo Electron Corporation, Waltham, MA, USA). The particle size and zeta potential of NA-FGF-N-2-HACC/CMCS MPs were determined by dynamic light scattering analysis (Nano-ZS90, Malvern Panalytical Limited, Almelo, Holland, MI, USA). The morphology of the NA-FGF-N-2-HACC/CMCS MPs was observed by transmission electron microscopy (TEM) (S-4800, Hitachi, Tokyo, Japan).

### 2.6. Oral Stability of NA-FGF-N-2-HACC/CMCS MPs

To test the oral stability of NA-FGF-N-2-HACC/CMCS MPs, NA-FGF-N-2-HACC/CMCS MPs were dispersed in simulated gastric fluid (SGF), simulated bile fluid (SBF), and simulated intestinal fluid (SIF), respectively. After incubating in a shaker at 37 °C, the digesta of NA-FGF-N-2-HACC/CMCS MPs were collected, and the light transmittance was measured. Each measurement was performed in triplicate.

### 2.7. Safety of NA-FGF-N-2-HACC/CMCS MPs

To test the biocompatibility and biosafety of the prepared microspheres, the cell viability and the hemolysis rate were calculated separately using a microplate reader (SpectraMax190, Molecular Devices Corporation, Sunnywell, CA, USA). The cytotoxicity of NA-FGF-N-2-HACC/CMCS MPs against cells was determined using the CCK-8 kit. Cells were seeded in 96-well plates and incubated in a 37 °C incubator containing 5% CO_2_ for 24 h. The old culture media was discarded, and different concentrations of NA-FGF-N-2-HACC/CMCS MPs (0, 10, 25, 50, 100, 250, and 500 μg/mL) were added into the well. Cells were further incubated for another 24 h. After washing with PBS, 10 μL CCK-8 was added to each well and incubated for 2 h. The absorbance at OD_450_ nm was measured using a microplate reader three times, and the cell viability was calculated.

For hemolysis analysis, 1 mL of fresh rabbit blood was collected, diluted in 2 mL of PBS, centrifuged for 10 min at 1000 r/min, and diluted in 10 mL of PBS. Then, 0.8 mL of NA-FGF-N-2-HACC/CMCS MPs at different concentrations (0, 25, 50, 100, and 250 μg/mL) and 0.2 mL of diluted red blood cells were added and mixed well at a constant temperature of 37 °C for 60 min. Finally, it was centrifuged, and 0.2 mL of each supernatant was taken to measure the absorbance at 570 nm using a microplate reader. Furthermore, mice were injected intraperitoneally with NA-FGF-N-2-HACC/CMCS MPs at a dose of 50 mg/kg, and the blood glucose changes were measured within 10 h after administration.

### 2.8. Pharmacodynamic Analysis of NA-FGF-N-2-HACC/CMCS MPs

The diabetic mice were randomly divided into three groups of five mice each. The first group of mice were injected intraperitoneally with saline; the second group of mice were injected intraperitoneally with free NA-FGF; and the third group of mice were injected intraperitoneally with NA-FGF-N-2-HACC/CMCS MPs. The injected dose was 1 mg/kg. Mice were injected continuously for 4 weeks, and the blood glucose values were measured every week.

### 2.9. Analysis of the Blood Glucose Regulation by NA-FGF-N-2-HACC/CMCS MPs via Oral Administration

The diabetic mice were randomly divided into three groups of five mice each. The first group of mice were injected intraperitoneally with saline; the second group of mice were injected intraperitoneally with NA-FGF; and the third group of mice were administered intragastrically with NA-FGF-N-2-HACC/CMCS MPs. The administration dose was 1 mg/kg. After 1, 2, 3, 4, and 5 h post-administration, blood was collected from the tail vein, and the blood glucose changes were detected by a glucometer.

### 2.10. In Vitro and In Vivo Release Capacity of NA-FGF-N-2-HACC/CMCS MPs

To test the in vitro sustained-release effect of the prepared microspheres, the NA-FGF-N-2-HACC/CMCS MPs were dispersed into 1.5 mL of PBS (pH 7.2–7.4). After incubating at different time intervals, the suspension was centrifuged, and the supernatant was collected to determine the release of NA-FGF from NA-FGF-N-2-HACC/CMCS MPs by UV-Vis spectrophotometry (U-5100, Tokyo, Japan). For the investigation of the in vivo sustained-release effect of NA-FGF-N-2-HACC/CMCS MPs, the diabetic mice were injected intraperitoneally with NA-FGF and NA-FGF-N-2-HACC/CMCS MPs, respectively, and the injection dose was 1 mg/kg. Each group was divided into single injections and multiple injections (once a day and continuous injections for one week). The blood glucose changes at different time intervals were monitored.

### 2.11. Statistical Analysis

Statistical analysis was performed using the Student’s *t*-test and analyzed by SPSS Statistics 19 software. All experimental data were expressed as mean ± standard error, and the comparison between means was performed by variance analysis. * *p* < 0.05; ** *p* < 0.01; *** *p* < 0.001; **** *p* < 0.0001.

## 3. Results

### 3.1. Detection and Biological Activity of the NA-FGF

**Figure 1:**
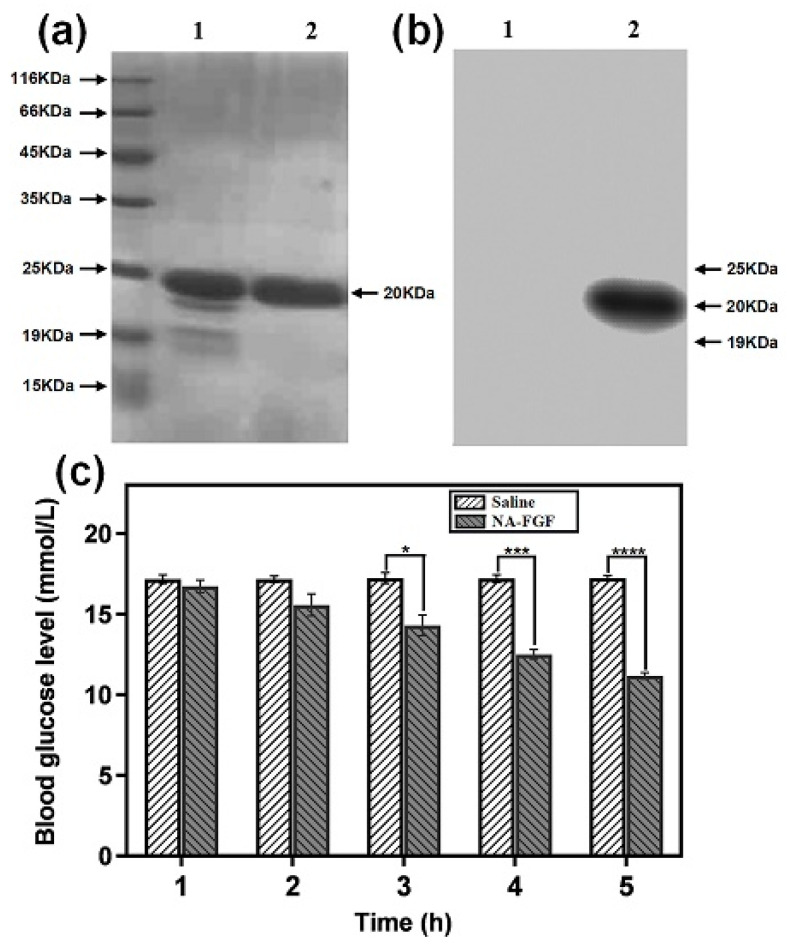
(**a**) SDS-PAGE analysis of the NA-FGF produced by strain DNZY1. Lane 1 indicated the primary purification and Lane 2 represented the secondary purification. (**b**) Western blot analysis of the NA-FGF. Lane 1 indicated the primary purification and Lane 2 represented the secondary purification. (**c**) Blood glucose levels of diabetic mice after being injected with the saline and the NA-FGF, respectively. Error bars represent the standard deviation of three experiments (*n* = 5 for each group) (* *p* < 0.05; *** *p* < 0.001; **** *p* < 0.0001).

The NA-FGF protein was expressed in the *E. coli* expression system, and the purity of NA-FGF was characterized by SDS-PAGE analysis. As shown in Figure 1a, compared with the primary purification (Lane 1), the band appeared at 20 KDa in Lane 2 with no spurious band, suggesting the successful preparation and purification of NA-FGF. In addition, western blotting analysis showed that the NA-FGF from strain DNZY1 was specific and could react with the anti-FGF-21 antibody (Figure 1b). Since NA-FGF has a significant effect on the regulation of blood glucose levels, the activity of the purified NA-FGF was detected using the diabetic mouse model. Figure 1c showed no hypoglycemic effect in the saline injection group. Conversely, a significant hypoglycemic effect could be observed in the NA-FGF injection group in a time-dependent manner, and the blood glucose values of mice decreased from 17.07 ± 0.29 mmol/L to 11.17 ± 0.21 mmol/L after 5 h, indicating that NA-FGF could reduce blood glucose levels in diabetic mice with a certain biological activity.

### 3.2. Preparation and Optimization of NA-FGF-N-2-HACC/CMCS MPs

**Figure 2:**
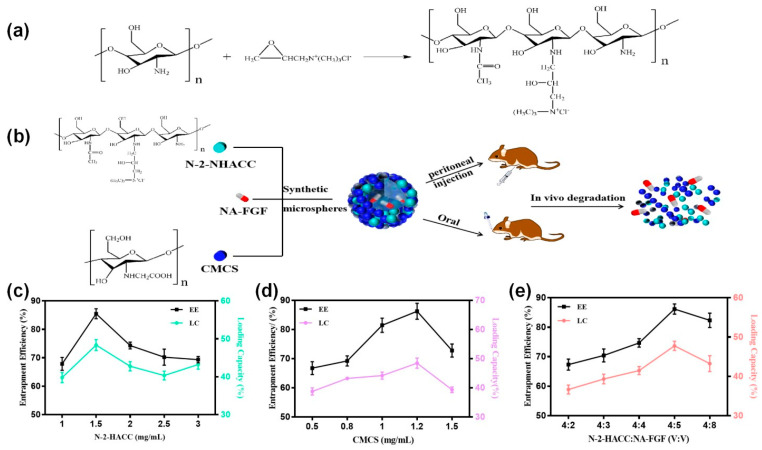
(**a**) Synthesis mechanism of N-2-HACC. (**b**) Schematic representation of NA-FGF-N-2-HACC/CMCS MPs used for injection and oral administration. Effect of (**c**) N-2-HACC concentration; (**d**) CMCS concentration; and (**e**) N-2-HACC:NA-FGF (*v*:*v*) on EE and LC of NA-FGF-N-2-HACC/CMCS MPs.

First, a water-soluble chitosan derivative, N-2-HACC, was synthesized in the laboratory (Figure 2a). NA-FGF-N-2-HACC/CMCS MPs were then prepared by the ion cross-linking method (Figure 2b). To obtain the particle size and morphology of the microspheres, the preparation conditions were optimized, and the final conditions of the prepared microspheres were determined by encapsulation efficiency and loading capacity. The optimal N-2-HACC concentration was 1.5 mg/mL, the optimal CMCS concentration was 1.2 mg/mL, and the volume ratio of N-2-HACC and NA-FGF was 4:5 (Figure 2c–e). The encapsulation efficiency and loading capacity of NA-FGF-N-2-HACC/CMCS MPs were 90.1 ± 2.6% and 48.6 ± 2.4%, respectively (Table 1).

### 3.3. Characterization of NA-FGF-N-2-HACC/CMCS MPs

**Figure 3:**
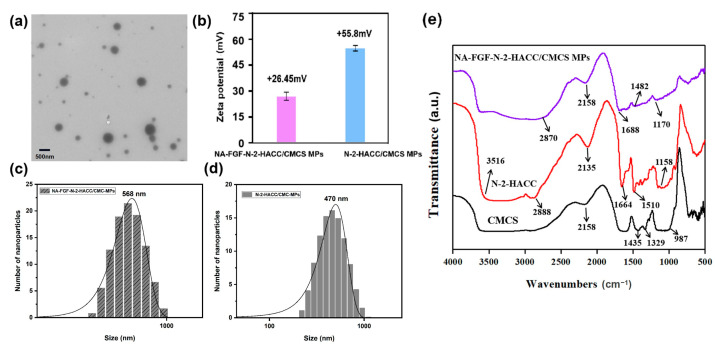
(**a**) TEM image of NA-FGF-N-2-HACC/CMCS MPs. (**b**) Zeta potential of NA-FGF-N-2-HACC/CMCS MPs and N-2-HACC/CMCS MPs. (**c**,**d**) Size distributions of NA-FGF-N-2-HACC/CMCS MPs and N-2-HACC/CMCS MPs, respectively. (**e**) FTIR spectra of N-2-HACC, CMCS, and NA-FGF-N-2-HACC/CMCS MPs.

Based on the successful preparation of NA-FGF-N-2-HACC/CMCS MPs, the morphology was characterized by TEM. The TEM image revealed that the NA-FGF-N-2-HACC/CMCS MPs were spherical in shape with regular and smooth morphology (Figure 3a). The zeta potential of the NA-FGF-N-2-HACC/CMCS MPs was +26.45 mV, and the zeta potential of the N-2-HACC/CMCS MPs was +55.8 mV (Figure 3b). Figure 3c,d showed the average particle sizes of NA-FGF-N-2-HACC/CMCS MPs and N-2-HACC/CMCS MPs were 568 nm (PDI = 0.331) and 470 nm (PDI = 0.331), respectively, indicating a slight increase in particle size after encapsulating the NA-FGF. Subsequently, FTIR spectroscopy was applied to characterize the interaction between N-2-HACC and CMCS (Figure 3e). In the FTIR spectra of N-2-HACC, the stretching vibration absorption peaks assigned to N–H and O–H appeared at 3516 cm^−1^, the stretching vibration peak at 1510 cm^−1^ was assigned to –NH_2_, and the stretching vibration peaks assigned to saturated CH (–CH_2_ and –CH_3_) appeared at 2888 cm^−1^. For FTIR spectra of CMCS, the telescopic vibration peaks of –O– and C–O appeared at 987 cm^−1^, and the characteristic peaks at 1329 cm^−1^ and 1435 cm^−1^ were identified as the asymmetric and symmetrical absorption peaks of –COO–. In comparison with N-2-HACC and CMCS, the FTIR spectra of NA-FGF-N-2-HACC/CMCS MPs changed accordingly. The characteristic absorption peak of –COOH appeared at 1482 cm^−1^, and the characteristic peak of -NH_2_ variable angle and NH^3+^ was presented at 1688 cm^−1^. These results revealed the synthesis of N-2-HACC and CMCS and the successful construction of NA-FGF-N-2-HACC/CMCS MPs.

### 3.4. Oral Stability Analysis of NA-FGF-N-2-HACC/CMCS MPs

**Figure 4:**
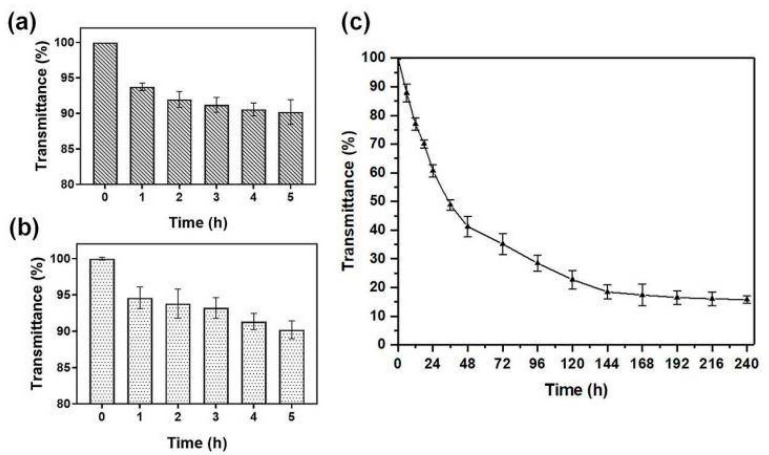
(**a**) Light transmittance of NA-FGF-N-2-HACC/CMCS MPs in simulant SGF. (**b**) Light transmittance of NA-FGF-N-2-HACC/CMCS MPs in simulant SBF. (**c**) Light transmittance of NA-FGF-N-2-HACC/CMCS MPs in simulant SIF.

The environment in the stomach, such as a low pH and the presence of substantial pepsin, is not conducive to the effective delivery and function of therapeutic drugs. The pH of human and animal gastric juice is generally between 1 and 1.5, and the drug is easily destroyed in the stomach. Therefore, the stability of NA-FGF-N-2-HACC/CMCS MPs in the simulated gastric environment was investigated. Figure 4a,b showed that the light transmittance of NA-FGF-N-2-HACC/CMCS MPs remained above 90% after exposure to simulated gastric fluid (SGF) and simulated bile fluid (SBF) for 4 h, respectively, demonstrating that NA-FGF-N-2-HACC/CMCS MPs could resist gastric juice and overcome pepsin degradation. When the prepared microspheres passed smoothly through the gastric environment, they also had a good tolerance to bile and could avoid the corrosion of bile salts. The main components of artificial intestinal fluid were phosphate and trypsin. To verify whether NA-FGF-N-2-HACC/CMCS MPs could overcome the degradation of trypsin as well as intestinal stability, the transmittance of NA-FGF-N-2-HACC/CMCS MPs in simulated intestinal fluid (SIF) for 240 h was also tested to determine intestinal stability. Generally, the drug is absorbed by the intestine within 6 h. Figure 4c revealed that the NA-FGF-N-2-HACC/CMCS MPs maintain more than 70% light transmittance within 12 h and can be completely degraded after 240 h, so the NA-FGF-N-2-HACC/CMCS MPs can have enough time to exert their efficacy during this period. This proves that NA-FGF-N-2-HACC/CMCS MPs are stable in the intestinal fluid. The results suggested that NA-FGF-N-2-HACC/CMCS MPs have great biostability and biological activity to overcome gastric and intestinal damage and effectively resist the degradation of trypsin, which proved that they were suitable for oral administration.

### 3.5. Biosafety Analysis of NA-FGF-N-2-HACC/CMCS MPs

**Figure 5:**
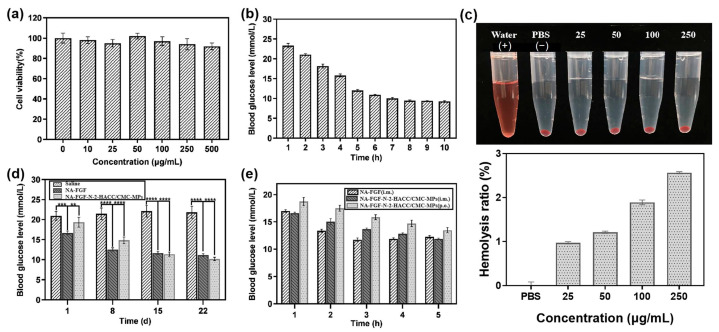
(**a**) Cell viability of ST cells after incubation with N-2-HACC/CMCS MPs. (**b**) The level of blood glucose in mice after being treated with a high-dose injection of NA-FGF-N-2-HACC/CMCS MPs (50 mg/kg) (*n* = 5). (**c**) Hemolysis ratio of NA-FGF-N-2-HACC/CMCS MPs. (**d**) Blood glucose changes after mice were treated with NA-FGF and NA-FGF-N-2-HACC/CMCS MPs, respectively (*n* = 5 for each group). (**e**) The blood glucose level of mice after treated with NA-FGF-N-2-HACC/CMCS MPs through oral and injection administration, respectively. Error bars represent the standard deviation of three experiments (*n* = 5 for each group) (** *p* < 0.01; *** *p* < 0.001; **** *p* < 0.0001).

Before investigating the blood glucose regulation ability of the prepared microspheres, the biosafety and biocompatibility of NA-FGF-N-2-HACC/CMCS MPs were evaluated. Negligible cytotoxicity could be detected after cells were co-incubated with NA-FGF-N-2-HACC/CMCS MPs (Figure 5a). Mice injected with a high dose of NA-FGF-N-2-HACC/CMCS MPs (50 mg/kg) were also employed to further assess the biosafety of NA-FGF-N-2-HACC/CMCS MPs. Figure 5b suggests that after the high-dose injection of NA-FGF-N-2-HACC/CMCS MPs, the blood glucose level of mice decreased steadily from 1 to 5 h, and there was no sudden decrease in blood glucose in a short period. These results showed negligible side effects, such as hypoglycemia, could be observed after high-dose injection of the NA-FGF-N-2-HACC/CMCS MPs, revealing that NA-FGF-N-2-HACC/CMCS MPs possessed great biosafety and excellent hypoglycemic effect. Additionally, the hemolysis rate of NA-FGF-N-2-HACC/CMCS MPs was calculated to be less than 5%, which meets the requirements of medical experimental materials (Figure 5c).

### 3.6. Pharmacodynamic Analysis of NA-FGF-N-2-HACC/CMCS MPs

To assess the in vivo efficiency of NA-FGF-N-2-HACC/CMCS MPs for diabetes treatment, the intraperitoneal injection of physiological saline and NA-FGF was used as a control group, and the intraperitoneal injection of NA-FGF-N-2-HACC/CMCS MPs was employed as the experimental group. The injected dose was 1 mg/kg. Mice were injected continuously for 4 weeks, and the blood glucose values were measured every week. As shown in Figure 5d, there were no significant changes in blood glucose levels in the group of physiological salines within 4 weeks. In contrast, both the NA-FGF injection group and the NA-FGF-N-2-HACC/CMCS MPs injection group reduced the blood glucose level. It could be noted that the rapid decline of blood glucose levels in the first 8 days of the NA-FGF injection group easily led to hypoglycemia in mice. However, the decline of blood glucose levels in the NA-FGF-N-2-HACC/CMCS MPs group changed steadily. On this basis, it indicated that the NA-FGF-N-2-HACC/CMCS MPs exhibited a better hypoglycemic effect with fewer side effects than the free NA-FGF.

### 3.7. Blood Glucose Regulation of NA-FGF-N-2-HACC/CMCS MPs via Oral Administration

Since direct oral administration of NA-FGF is easy to degrade in the gastrointestinal tract and cannot exert a significant effect on lowering blood glucose regulation, N-2-HACC/CMCS particles have been proven to be an excellent delivery carrier to encapsulate proteins and drugs for in vivo delivery. Therefore, the hypoglycemic effect of NA-FGF-N-2-HACC/CMCS MPs through oral administration was then examined. The result in Figure 5e suggested that oral NA-FGF-N-2-HACC/CMCS MPs still possessed the glucose-regulation effect, while the hypoglycemic effect was not as obvious as that of the injection group. This may be because the complex mucosal microenvironment, such as mucosal clearance and gastric acid degradation, partly affected the glucose-lowering effect of oral NA-FGF-N-2-HACC/CMCS MPs. Furthermore, it should be noted that in the first 3 h post-treatment, the injection of free NA-FGF could play a rapid role in reducing blood sugar, and the blood glucose level rebounded after 4 h due to the decrease in NA-FGF. However, either oral or injected NA-FGF-N-2-HACC/CMCS MPs could regulate blood glucose levels for a long time, and there will be no disordered blood glucose levels caused by the sharp decline in blood glucose levels after treatments.

### 3.8. In Vitro and In Vivo Release Analysis of the NA-FGF-N-2-HACC/CMCS MPs

**Figure 6:**
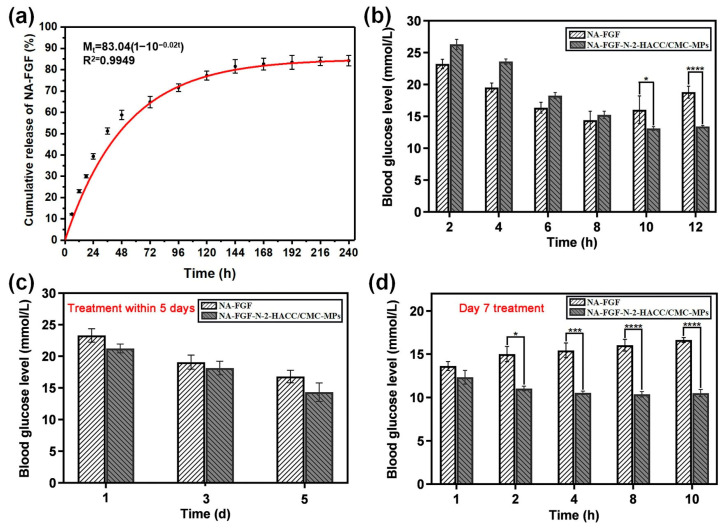
In vitro and in vivo release behavior of NA-FGF-N-2-HACC/CMCS MPs: (**a**) In vitro accumulative release of NA-FGF from NA-FGF-N-2-HACC/CMCS MPs; (**b**) Blood glucose level changes after mice were singly administered with NA-FGF-N-2-HACC/CMCS MPs; (**c**) Blood glucose level changes after mice were injected multiple times at 1, 3, and 5 days; (**d**) Blood glucose level changes after mice were injected multiple times on day 7. Error bars represent the standard deviation (*n* = 5 for each group) (* *p* < 0.05; *** *p* < 0.001; **** *p* < 0.0001).

The limited release of NA-FGF is a significant safety feature for its in vivo application. Thus, the release property of NA-FGF-N-2-HACC/CMCS MPs was investigated. The in vitro release capacity of NA-FGF-N-2-HACC/CMCS MPs was assessed in PBS (pH = 7.4). As shown in Figure 6a, the in vitro release curves of NA-FGF-N-2-HACC/CMCS MPs exhibited a rapid release of NA-FGF from NA-FGF-N-2-HACC/CMCS MPs in the initial 48 h. After 48 h, the release of NA-FGF gradually increased from 58.76% to 81.56%. The accumulative release of NA-FGF in NA-FGF-N-2-HACC/CMCS MPs reached 84.21% after 240 h. It could be seen that the long-term release of NA-FGF from NA-FGF-N-2-HACC/CMCS MPs is beneficial for being absorbed and utilized by the organism, demonstrating the great sustained release ability of N-2-HACC/CMCS MPs for effective drug-release and disease treatments.

Subsequently, the in vivo release ability of NA-FGF-N-2-HACC/CMCS MPs was studied via both single injections and multiple injections (once a day for a week). The result in Figure 6b showed that a single injection of NA-FGF-N-2-HACC/CMCS MPs could realize a sustained release of NA-FGF with long-term regulation of blood glucose levels. In contrast, the injection of free NA-FGF could only be maintained for 8 h, and the blood glucose value gradually returned to the pre-treatment level. Similarly, the multiple injections of NA-FGF-N-2-HACC/CMCS MPs could also maintain the normal blood glucose level range after one week of continuous treatments. Figure 6c,d show the effects of blood glucose levels in mice given multiple injections. Figure 6c shows that NA-FGF and NA-FGF-N-2-HACC/CMCS MPs can equally decrease blood glucose during the first 5 days. Figure 6d shows that from day 7, the blood glucose level of the NA-FGF-treated group began to rise seriously, indicating that the free NA-FGF could only play a role for a short period after injection. The blood glucose level of the NA-FGF-N-2-HACC/CMCS MPs-treated group mice remained stable in the normal level range, which showed that NA-FGF-N-2-HACC/CMCS MPs had a long-term slow-release effect. It may be because the NA-FGF-N-2-HACC/CMCS MPs injected multiple times in the abdominal cavity can be absorbed through the mesenteric vein of the abdomen and then gradually enter the systemic circulation. The NA-FGF-N-2-HACC/CMCS MPs release NA-FGF under physiological conditions to achieve the effect of maintaining stable blood glucose levels for a long time. These results demonstrated that the NA-FGF-N-2-HACC/CMCS MPs have a longer-term effect on regulating blood glucose compared with that of the free NA-FG.

## 4. Discussion

Insulin injection is currently one of the most commonly used approaches for the treatment of diabetes mellitus. However, the subcutaneous injection of insulin is easy to peak at night, making patients produce hypoglycemia. Although some methods have been developed to overcome the defects caused by conventional insulin injection, such as micro-infusion automated systems [38], we also need to develop ways that are more acceptable and convenient for patients. Subcutaneous injection has weak control over blood glucose fluctuations, and frequent injection is accompanied by pain, trauma, local tissue necrosis, and nerve injury [39]. In addition, long-term injections are prone to causing disorders of fat metabolism and fat hyperplasia. As a kind of polypeptide, the direct oral administration of insulin will be easily degraded and inactivated by gastrointestinal digestive enzymes, and the in vivo bioavailability of insulin is less than 2% [40]. The large molecular weight of insulin also makes it difficult to pass the absorption barrier of the intestinal tract. In recent years, numerous studies have focused on improving the oral bioavailability of insulin and overcoming the poor compliance caused by subcutaneous injections. Although oral treatment of diabetes has made significant progress in achieving convenience and effectiveness, different insulin-based agents still face great challenges in toxicology, poor stability, and individual variability in absorption.

FGF-21 analogs can regulate blood glucose, decrease blood lipids, and improve insulin resistance. On this basis, FGF-21 analogs with a longer-lasting hypoglycemic effect obtained by the bio-fermentation and purification of lipocin gene-engineered strain DNZY1 have been developed. However, such proteins are at risk of poor stability and a short half-life, which directly affect their clinical therapeutic effects. Microspheres and nanoparticles have been used as effective ways of delivering drugs due to their high drug-loading capacity, great sustained release performance, high structural stability, and excellent biocompatibility. Among them, chitosan derivative-based microspheres constructed by the ion-crosslinking method exhibited great promise. Herein, FGF-21 analogs (NA-FGF) that were bio-fermented and purified from strain DNZY1 with longer-lasting hypoglycemic effects were used as the diabetes treatment drug, and N-2-HACC/CMCS MPs were employed as the carrier to construct the NA-FGF-N-2-HACC/CMCS MPs. The prepared microspheres have great properties such as excellent biosafety, effective drug-delivery ability, and long-lasting release capacity. The NA-FGF-N-2-HACC/CMCS MPs were prepared by complex coacervation, and the morphology of the particles was intact and rounded. The average particle size was 568 nm. The particle size of microspheres is an important factor in their passing through the mucosal surface, and it is easier to cross the mucosal barrier when the particle size is less than 1 μm. After the NA-FGF-N-2-HACC/CMCS MPs entered the body, the microspheres were decomposed and released the therapeutic NA-FGF. The injection administration of NA-FGF-N-2-HACC/CMCS MPs can avoid the first-pass effect and quickly deliver the drug to the blood circulation system. In addition, NA-FGF-N-2-HACC/CMCS MPs can also be administered through the nasal cavity and lung [41]. Although injection administration is commonly used in clinical practice, this approach requires repeated multiple injections for low patient adaptability, is invasive, and is prone to causing wound infection [42]. Oral administration has been the most acceptable route for patients. Owing to the unique properties of chitosan and chitosan derivatives, such as great bio-adhesion and mucosal penetration, the NA-FGF-N-2-HACC/CMCS MPs can significantly enhance the absorption and bioavailability of NA-FGF in vivo. The results of our study showed that the NA-FGF-N-2-HACC/CMCS MPs administered orally could also regulate the blood glucose level, though the effect was not as significant as the injection. The specific mechanism of the therapeutic effect of oral NA-FGF-N-2-HACC/CMCS MPs in vivo still needs to be further explored.

## 5. Conclusions

In summary, biocompatible NA-FGF-N-2-HACC/CMCS MPs with a significant hypoglycemic effect were prepared and characterized. We demonstrated that NA-FGF-N-2-HACC/CMCS MPs presented uniform, smooth spherical particle sizes with low cytotoxicity, good stability, and biocompatibility. In vivo trials have revealed the great safety and sustained-release effects of NA-FGF-N-2-HACC/CMCS MPs. In addition, both injection and oral administration reduced the blood glucose level in diabetic mice. The NA-FGF-N-2-HACC/CMCS MPs improved the bioavailability of NA-FGF during the process of blood glucose regulation, extended the time of action in vivo, and reduced the frequency of administration. Although we hope to change the traditional way of administration from injection to oral administration, the effect still needs further improvement and has laid the foundation for future research. We believe that the N-2-HACC/CMCS-based delivery systems may have promising potential in the fields of biological and biomedical research.

## Figures and Tables

**Table 1:** Results of orthogonal experimental design.

N-2-HACC Concentration (mg/mL)	CMCS Concentration (mg/mL)	N-2-HACC:NA-FGF (*v*:*v*)	EE (%)	LC (%)
1.5	0.8	4:4	71.1 ± 2.2	40.2 ± 1.1
1.5	1.0	4:8	72.4 ± 1.8	39.8 ± 1.2
1.5	1.2	4:5	89.4 ± 2.8	48.8 ± 1.4
2.0	0.8	4:8	82.2 ± 1.4	43.4 ± 1.5
2.0	1.0	4:5	80.4 ± 1.6	42.7 ± 1.8
2.0	1.2	4:4	84.6 ± 2.5	44.4 ± 1.3
3.0	0.8	4:5	76.7 ± 2.0	38.8 ± 1.0
3.0	1.0	4:4	79.5 ± 2.4	41.1 ± 1.2
3.0	1.2	4:8	80.6 ± 2.1	37.8 ± 0.85

## Data Availability

The data presented in this study are available on request from the corresponding author.
